# Challenges and Opportunities for Deliberative Processes for Healthcare Decision-Making: Comment on "Evidence-Informed Deliberative Processes for Health Benefit Package Design – Part II: A Practical Guide"

**DOI:** 10.34172/ijhpm.2022.7458

**Published:** 2022-08-16

**Authors:** Kenneth Bond

**Affiliations:** Institute of Health Economics, Edmonton, AB, Canada.

**Keywords:** Deliberation, Deliberative Processes, Health Technology Assessment, Legitimacy, Priority Setting, Stakeholder Involvement

## Abstract

The second edition of the practical guide for evidence-informed deliberative processes (EDPs) is an important addition to the growing guidance on deliberative processes supporting priority setting in healthcare. While the practical guide draws on an extensive amount of information collected on established and developing processes within a range of countries, EDPs present health technology assessment (HTA) bodies with several challenges. (1) Basing recommendations on current processes that have not been well-evaluated and that have changed over time may lead to weaker legitimacy than desired. (2) The requirement for social learning among stakeholders may require increased resourcing and blur the boundary between moral deliberation and political negotiation. (3) Robust evaluation should be based on an explicit theory of change, and some process outcomes may be poor guides to overall improvement of EDPs. This comment clarifies and reinforces the recommendations provided in the practical guide.

## Introduction

 In the last decade, there has been an increasing interest in and discussion about deliberative processes to support healthcare priority setting decisions. A recently published report by an HTAi-ISPOR task force^1^ describes a set of minimum features necessary for deliberative processes in health technology assessment (HTA) and provides a checklist and practical recommendations for those seeking to establish, improve or evaluate deliberative processes within HTA. Others have sought to provide practical guidance for deliberative processes by articulating the “supporting actions” that operationalize principles such as transparency, inclusivity, and impartiality.^2^ The recent article by Oortwijn et al on “evidence-informed deliberative processes,”^3^ (EDPs) and its companion^4^ (also published in this journal) are important additions to the developing body of research and guidance regarding deliberative processes in healthcare priority setting. The guidance in the two papers, which is a helpful summary and clarification of information contained in their Practical Guide for EDPs,^5^ is intended primarily for staff in HTA organizations (at various distances from government) who are responsible for establishing, operating and managing, or monitoring and evaluating EDPs. The aim of the guidance is to provide those staff with examples of and recommendations about how to operationalize the principles of “accountability for reasonableness,” arguably the dominant procedural principles for deliberative processes in HTA globally, with the aim of improving the legitimacy of the decisions of HTA bodies through stakeholder involvement, evidence-informed evaluation, transparency, and mechanisms for appeal. Here I comment briefly on three aspects of Part II of the guidance^3^ that have important implications for how the guidance is understood and used: viewing current EDPs as “best practice,” conceptualizing the aim of deliberation in terms of social learning among a broad group of stakeholders, and the identification and use of outcomes for monitoring and evaluation.

## Viewing Current EDPs as “Best Practice”

 It is common within the field of HTA research to label established processes as “best practice,” and, while the authors see their guide as “inspiration” for those looking to improve or establish EDPs, I want to sound a note of caution when inferring recommendations for EDPs from observed practices. Many of the mature or “inspirational” EDPs used as examples have changed over time in response to the organizational, political, economic, and other pressures of their respective health systems. The extent to which these changes have strengthened or weakened the efficiency and legitimacy of priority setting decisions has rarely been examined. As others have noted,^6^ we have a poor understanding currently of how factors other than the evidence being considered influence decisions, and how fair and effective HTA processes can be constructed in light of these potential influences. Accepting current processes as “best practice” without understanding these factors can have important consequences. For example, the EDP guidance recommends that advisory committees have 10-15 members. The sizes of committees vary, and we do not have a good understanding of why different committees have the number of members they do, and how that difference is related, if at all, to the robustness of decision-making, perceptions of fairness, or other desirable outcomes or important principles. This lack of understanding is reflected in the need expressed by HTA agencies for guidance specifically in this area.^7^

 Evaluating the legitimacy of established EDPs has fallen primarily to interested and motivated academic researchers. Gathering the documentation that is required to conduct these evaluations relies on close cooperation with HTA bodies, and a degree of transparency that HTA bodies may find challenging. Researchers who have assembled the documentation needed to assess EDPs overseen by England’s National Institute for Health and Care Excellence^8,9^ have concluded that changes the organization has made to its procedural and substantive principles has undermined the fairness and legitimacy of its decision-making.^9^ Other HTA bodies have made similar changes. For example, the Canadian Agency for Drugs and Technologies in Health has made changes to its pharmaceutical reimbursement recommendations framework^10^; yet, to my knowledge, no assessment has been made of these changes, and there is little information available to explain the overall approach to and rationale for having made those changes.

 Many of the recommendations regarding “best practice” are based on EDPs in high-income countries. While this is understandable, Oortwijn and colleagues have gained important insights through their research and experience in working with low- and middle-income countries (LMICs). Given the desire on the part of HTA agencies to find efficiencies in deliberative processes, I would like to highlight the possibility of high-income countries looking to LMICs for inspiration — a process sometimes referred to as “reverse innovation.”^11^ The imperative to do more with less in LMICs, which may be particularly acute in healthcare, and the absence of established organizational structures and legislation that may constrain novel approaches, could provide an environment within which to find new possibilities for EDPs.

## The Aim of Stakeholder Involvement

 EDPs enhance the legitimacy of decisions through a deliberation among stakeholders to “identify, reflect and learn about the meaning and importance of values, informed by evidence about these values,” and improving the quality of decision-making by making it better informed,^4^ a process sometimes referred to as “social learning.” Stakeholders are defined broadly to include patients, the broader public, healthcare providers, purchasers, payers, policy makers, product manufacturers, and academic researchers. There are a number of tensions inherent in making the aim of deliberative processes social learning among this broad group of stakeholders.

 One point of tension is that this robust and broad ranging social learning may not be needed to secure the legitimacy of all decisions. EDPs view deliberation as a means to strengthening the legitimacy of decisions. While this learning may be important when making decisions about highly contested conditions or technologies, the more typical decisions of HTA bodies may neither require nor need this kind of interaction among stakeholders to satisfy sufficiently the conditions of accountability for reasonableness, which has as its focus the development and presentation of *facts, reasons, and principles *that would be found reasonable to those affected by the decision.This is a point about determining what is sufficient for legitimacy and not about settling controversy. If the careful, sensitive, and robust deliberation that constitutes social learning is not always required, then taking social learning as the main aim of deliberation (as a means to strengthen legitimacy) may result in unnecessary time and resource use for health benefit package decisions. There are processes and techniques falling between consultation and participation, such as Delphi, that may help to address these challenges.^12^

 HTA agencies, generally speaking, are not striving to reach an ideal process to secure legitimacy, but seeking to find efficiencies and sufficiency in addressing the various perspectives. Individual HTA organizations will need to find the best balance of procedural principles and practical efficiencies for their context when constructing EDPs. Hence, there are two practical questions that might be helpfully answered by the EDP guidance: (1) How might we determine which decisions could benefit most from or require a deepened engagement oriented toward social learning to further the aims of legitimacy and when might other less robust forms of involvement be used? (2) What are the relevant knowledge and skills required to effectively support values-based conversations of this kind, and to reveal and handle the value conflicts and moral uncertainty that may arise?

 Given the location of EDPs at the intersection of democratic governance and the application of research-based knowledge, some users of the guidance might reasonably conclude from the authors’ description of stakeholder involvement that deliberative processes function like a legislature, with the corollary that the aim of deliberation is to settle value conflicts in a way that would be satisfactory to each stakeholder.^13^ If this were to happen, there is a subsequent risk that the activity of deliberation is organized as a negotiation of interests and the ethical challenges of benefit package decisions are transformed into challenges that are resolved politically. It should be remembered that, for those involved in EDPs, there is an obligation on the part of all those who contribute to and benefit from a health system based on shared savings to restrain their demands in order to maintain the viability of that system.^13^

## Further Developing Monitoring and Evaluation

 An evaluation of EDPs “…ideally informs the HTA body about any shortcomings in terms of how their processes are being implemented and/or its overall impact and why this may be so.”^3^ Despite its importance, evaluation and monitoring are not well described by HTA agencies.^3^ As HTA bodies strive to do better on this front, I want to highlight some potential challenges in evaluation that are raised by the complex social, political, and ethical aims of EDPs, and that ought to be taken up by the HTA community.

 Not mentioned by the authors in their article, but described by them in their guide, is the importance of developing a theory of change to “…explain how the implementation of an EDP is expected to achieve desired impacts, in terms of inputs and activities, outputs, outcomes, and more long-term impacts.”^5^ This is an important point, and seems much neglected by HTA bodies; those developing and evaluating EDPs would do well to accept this challenge. As was noted earlier, in their efforts to improve EDPs in terms of more proximate process outcomes, such as consistency in decision-making, timeliness, greater acceptability of decisions, and speedier implementation of or access to new technologies, well-established EDPs, may have become less transparent and accountable, thus weakening legitimacy. A well-developed theory of change may help to identify proximate actions that HTA bodies can take to improve practice, while avoiding moving in directions that undermine their desired goals. This point is especially important because techniques, such as multi-criteria decision analysis, which are intended to incorporate a broad range of values and improve consistency in decision-making, require trade-offs of this kind.

 According to the authors, the main aims of EDPs are securing or improving the reasonableness of decisions as perceived by stakeholders, and sharing perspectives and values to maximize understanding and support among stakeholders. How might this “reasonableness” and “maximized understanding” be assessed? Researchers have noted that there is a need to evaluate the reasonableness of both procedural and substantive principles within priority setting processes.^13^ Few, if any, HTA bodies provide sufficiently transparent and detailed records of deliberations to be able to monitor and assess the development of “coherence and mutual understanding” among committee members. What is the appropriate standard for this coherence and mutual understanding? For example, many committees use a consensus model for decision-making. While many committees describe this aspect of their decision-making in their terms of reference (or similar documentation), I am not aware of an HTA body that has provided an explicit rationale for the use of consensus or connected this model explicitly to the aims of EDPs, as has been attempted for ethics committees, for example.^14^ Better articulating the nature of the committees and their decisions will have important implications for how committees operate, such as the number of members and their expertise.

 HTA bodies must balance various competing values and actions to provide recommendations or decisions at the appropriate time for decision makers. To do so, gains on one feature of EDPs, for example, timeliness of outputs, may come at the expense of another, such as stakeholder involvement in input and deliberation. There are opportunity costs for structuring EDPs in one way rather than another, and not all agencies will choose to make the same trade-offs. Evaluations must be conducted carefully, and in detail, so as not to oversimply the complex considerations and competing values that have led to an EDP being set up in a particular way. In EDPs, as with many other things, context and details are important. As one of the guide’s authors has elsewhere concluded “…best practices that may work well in some countries might not necessarily work evenly well in other countries.”^15^

## Conclusion

 The updated practical guide on EDPs provides helpful examples and recommendations for those seeking to establish or improve deliberative processes to support the legitimacy of health benefit package decisions. In light of our current understanding of EDPs, I have pointed to some reasons for caution when looking to existing processes for inspiration, to some challenges with conceiving of deliberation in terms of social learning, and when identifying and selecting outcomes for evaluating and comparing EDPs. Nevertheless, it is important to acknowledge the complexity of ensuring legitimate decision-making and be respectful of the time, effort, and resources governments and HTA bodies devote to establishing and managing robust and meaningful EDPs, and to not be overly critical. Exercising appropriate caution, current processes can, indeed, inspire.

## Acknowledgements

 I would like to thank Professor Julia Abelson (McMaster University, ON, Canada) for helpful comments on an early draft of this commentary. I would also like to thank an anonymous reviewer for their thoughtful and constructive comments.

## Ethical issues

 Not applicable.

## Competing interests

 Author declares that he has no competing interests.

## Author’s contribution

 KB is the single author of the paper.
